# JFAR’s role in publishing believable research findings

**DOI:** 10.1186/1757-1146-6-49

**Published:** 2013-12-27

**Authors:** Karl B Landorf, Hylton B Menz, Alan M Borthwick, Mike J Potter, Shannon E Munteanu, Catherine J Bowen

**Affiliations:** 1 Lower Extremity and Gait Studies Research Program, Faculty of Health Sciences, La Trobe University, Bundoora, Victoria, Australia; 2 Faculty of Health Sciences, University of Southampton, Southampton, UK; 3 Department of Podiatry, Faculty of Health Sciences, La Trobe University, Bundoora, Victoria, Australia

## 

Health research published in journals provides an ever-increasing number of studies and trials that espouse the benefits of all sorts of interventions. But how do consumers of research, for example the readers of *Journal of Foot and Ankle Research (JFAR)*, know whether to believe the findings of research articles that they read? Clearly, health journals have an unambiguous responsibility to support good quality research that provides believable findings. Equally, they also have a responsibility to filter out poor quality research that provides findings that are not believable.

JFAR steadfastly supports good quality research and the editorial team (with the assistance of the peer-reviewers) work conscientiously to publish only those studies that are of the highest quality; that is, studies with believable findings. While this is not always easy, and the goal posts that define quality of research are constantly being repositioned, the editors strive to bring the readers of JFAR the best foot and ankle research. Unfortunately, not all studies use perfect methods, so if a study does have limitations and is published, then the editors are careful to ensure that the authors clearly acknowledge the limitations in their articles, affording readers an insight into the pitfalls of the study.

To assist the editors in this task, there is a burgeoning array of guidelines and recommendations for the conduct and reporting of different study designs. For example, there are the CONsolidated Standards Of Reporting Trials (CONSORT) Statement [1] and the Preferred Reporting Items for Systematic Reviews and Meta-Analyses (PRISMA) Statement [2]. Each guideline has been developed to ensure that important aspects of a trial or review are reported by the authors. To assist in this process, there is a checklist for each item in the guideline, which covers key aspects of a study (e.g. blinding, sample size determination and statistical analysis) that authors should report to ensure that they have included essential pieces of information, which ultimately makes the study’s findings more believable. That is, it prevents authors from deceiving the reader, by concealing that they did not do something they should have, or equally bad, done something that they should not have.

As an added bonus to readers of such articles, if guidelines like CONSORT and PRISMA are followed they invariably make an article less ambiguous and much easier to understand what has occurred in the study. The developers of such guidelines are experts, with most having decades of highly active experience researching and practicing these study designs. They are also generous individuals that have provided the guidelines in consumer friendly packages that are freely available. Potential users of these guidelines can easily access them through the well-developed CONSORT [3] and PRISMA [4] websites. Further to the guidelines mentioned above, there are many others for different study designs, for example; the Guidelines for Reporting Reliability and Agreement Studies (GRRAS) [5] and the Strengthening the Reporting of Observational Studies in Epidemiology (STROBE) statement [6,7]. Authors are recommended to consult these where appropriate and an excellent resource when writing manuscripts is that of the Enhancing the QUAlity and Transparency Of health Research (EQUATOR) Network [8], which is a virtual one-stop shop for guidelines that are relevant to publishing in health science journals. The EQUATOR Network includes guidelines for both quantitative and qualitative research.

JFAR, as a BioMed central journal, adheres to these guidelines and authors of studies that use these study designs are obliged to include such checklists upon submission of their article. Few foot and ankle journals ensure this or even worse, do not even recommend this. However, JFAR is setting the benchmark in this area because the editors believe that it leads to the highest possible quality research articles for its readers, and consequently the most believable findings will be presented in JFAR.

Furthermore, if clinical trials – where participants receive an intervention that is evaluated – are considered in more detail, JFAR also demands that authors must register their trial with a recognised clinical trial registry. Ideally, clinical trials should be registered prior to recruitment. Up to now, JFAR has accepted clinical trials that have been registered with a recognised clinical trial registry after the first participant has been recruited. However, from now on, JFAR will only accept clinical trials that are registered prior to recruitment. Why is this important? To begin with, it means that all clinical trials can be tracked and the results of clinical trials are less likely to be *not* published, which occurs more frequently in ‘non-significant trials’ (where no difference is detected between interventions). However, an equally important reason is that it prevents investigators changing their protocol or analysis in light of their findings if they unwisely assess the results prior to the completion of the trial, or once they have evaluated the results but before publication (often referred to as over-analysing the data or “cherry-picking” the results, which results in bias). By default, this means that investigators have to commit to their protocol and analysis, thus keeping them honest, which again leads to more believable findings. There are many recognised clinical trial registries and a list of recommended registries can be found on the *International Committee of Medical Journal Editors* website (http://www.icmje.org/publishing_j.html).

With all of this information in mind, two key points need to be considered. Firstly, consumers of articles that are published in JFAR should be assured that they are reading articles that present findings from high quality research. Secondly, potential authors of research manuscripts being submitted to JFAR need to adhere to the standards set by the journal; that is, they must read the guidelines for authors and carefully follow them when preparing their manuscripts. The journal’s guidelines are clear (http://www.jfootankleres.com/authors/instructions/research) and it is now, more than ever, *not* acceptable for authors to plead ignorance. As the impact of JFAR continues to increase, the editors are receiving more and more manuscripts to be considered for publication. This allows the editors more freedom to choose only the best quality research articles. If a manuscript is poorly written at the outset – for example, it has not adhered to the journal’s guidelines – then it has a much greater chance of rejection. Currently, JFAR rejects over 40% of the manuscripts submitted to it, and this figure is rising as the journal becomes more popular. For the best health journals in the world, such as *New England Journal of Medicine* and *Lancet*, this figure is about 95%. This means that they only publish the highest quality research. While there are exceptions, this research will generally be the most believable and will have the most impact.

In conclusion, the editors of JFAR work hard to ensure that they provide a vehicle for the best quality foot and ankle research. While a small number of mistakes will undoubtedly be made and errors detected only after publication, readers can be assured that JFAR is trying to set the highest standards possible to foster a culture of believable research. We trust that readers of the journal can appreciate that such standards lead to better, more believable information for them. Some may view this as being elitist or exclusive, but if this is the price for believable research findings then that is a label the editors are prepared to wear. Conducting good research is not easy, and clear evidence can sometimes take years to compile (take the evidence for the ill-effects of tobacco smoking as a perfect example). Journals that follow an easy formula, where there are few checks and balances, ultimately provide a ‘fast food’ approach to health information – it may look fabulous and satiate ones appetite for knowledge easily, but it is not good for anyone in the long term. Professional journals have a responsibility to ensure that statements made in manuscripts can be supported by the study findings (i.e. good-quality evidence). This responsibility should not be viewed as being elitist or exclusive and in no way suppresses freedom of expression. Indeed, the fundamental premise of scientific publication is the reporting of empirically verifiable facts, which means believable information.
